# Refinement for Structured Concurrent Programs

**DOI:** 10.1007/978-3-030-53288-8_14

**Published:** 2020-06-13

**Authors:** Bernhard Kragl, Shaz Qadeer, Thomas A. Henzinger

**Affiliations:** 8 Microsoft Research Lab, Redmond, WA USA; 9 University of Southern California, Los Angeles, CA USA; 10 IST Austria, Klosterneuburg, Austria; 11 Novi, Seattle, USA

## Abstract

This paper presents a foundation for refining concurrent programs with structured control flow. The verification problem is decomposed into subproblems that aid interactive program development, proof reuse, and automation. The formalization in this paper is the basis of a new design and implementation of the Civl verifier.

## Introduction

We present a solution to the problem of proving that no execution of a concurrent program leads to a failure. This problem is equivalent to proving an arbitrary safety property on the program. In *deductive verification*, a proof system decomposes this verification problem into a set of *proof obligations* (or *verification conditions*), and discharging these obligations implies the correctness of the program. At its core, any proof system depends on *inductive invariants*, and, in general, these have to be supplied manually. Inventing an inductive invariant is especially challenging for concurrent programs, since it has to capture complicated relationships over the entire program state, across all concurrent computations. Thus, the main practical obstacle to deductive verification is a suitable interaction mode for the programmer to invent and supply the necessary proof hints. This paper develops and implements a systematic conceptual framework for supplying these proof hints on a structured representation of the concurrent program, specifically eliminating the need to write complex invariants on the low-level encoding of the program as a flat transition system.

The Civl verifier [18, 25] addresses the aforementioned challenge by advocating *layered refinement over structured concurrent programs*. Instead of the monolithic approach that requires the programmer to prove the safety of a program $\mathcal {P}$ directly, Civl allows the programmer to specify a chain of increasingly simpler programs $\mathcal {P}= \mathcal {P}_0, \mathcal {P}_1, \ldots , \mathcal {P}_n = \mathcal {P}'$ such that the safety of $\mathcal {P}_{i}$ implies the safety of $\mathcal {P}_{i-1}$ for all $i \in [1,n]$, thus transferring the safety obligation on $\mathcal {P}$ to $\mathcal {P}'$. The overall correctness of the program is established piecemeal by focusing on the invariant required for each refinement step separately. While the programmer does the creative work of specifying the chain of programs and the inductive invariant justifying each link in the chain, the tool automatically constructs the verification conditions underlying each refinement step.

The core principle of a layered refinement proof in Civl is *iterative program simplification* through two kinds of creative reasoning. First, the programmer must think about the primitive atomic actions used to specify a particular program $\mathcal {P}_{i}$ in the chain of programs. These atomic actions must be chosen to have useful commutativity properties which allow the tool to provably eliminate preemptions at many control locations in $\mathcal {P}_{i}$, thus creating large preemption-free execution fragments. Second, the programmer must think about the justification for the transformation of $\mathcal {P}_{i}$ into the next program $\mathcal {P}_{i+1}$. This transformation may be complex because (1) some of the variables in $\mathcal {P}_{i}$ may become irrelevant, (2) new variables may be needed for the primitive atomic actions in $\mathcal {P}_{i+1}$, and (3) the transformation may simplify complex control flow (branching, procedure calls, recursion, etc.) into a single step that executes an atomic action. This paper focuses on the necessary foundation and tool support for this second kind of creative reasoning.

We present our technique on an idealized yet general language RefPL, suitable for expressing structured parallelism, asynchronous computation, atomic actions of arbitrary granularity, and dynamically-scoped preemption-free code fragments. Using the design of RefPL and the formalization of its operational semantics, we present two technical contributions.

Our first contribution is a general proof rule for soundly abstracting a recursive RefPL program $\mathcal {P}$ into another RefPL program $\mathcal {P}'$ that hides subsets of global variables, local variables, procedures, and atomic actions in $\mathcal {P}$. Our proof rule goes beyond Civl in two ways. First, it provides the capability to hide local variables of procedures, specifically parameters, in addition to global variables. This capability allows us to replace a procedure with an atomic action with a smaller interface by hiding the extra parameters. Refinement proofs are simplified because it becomes easy to introduce local snapshots of global variables needed for specifications, pass these snapshots around as parameters to procedures, and finally recover the original interface by hiding these extra parameters. Second, unlike Civl our proof rule is capable of performing refinement proofs on arbitrarily recursive programs. Since hiding low-level details is the core principle of the layered refinement methodology, our proof rule contributes towards increasing the expressiveness of refinement proofs compared to Civl.

Our proof rule depends on invariants that constrain the reachable states of the program. Our second contribution, an aid to our refinement rule but also independently useful, is a new specification idiom called *yield invariants*—named, parameterized, and interference-free invariants that can be called in parallel with ordinary procedures to soundly constrain the interference possible at yields within the called procedure. Since a yield invariant is named, its definition is separate from its invocation, thereby allowing proofs of interference-freedom to be performed once and reused for each call site. Since it is parameterized, it can be specialized to the needs of a call site by passing suitable input parameters.

Reasoning with yield invariants becomes difficult in concurrent programs when the absence of interference must be justified using facts referring to local variables of different procedures executing in different threads. The alternative of using global ghost variables that have the same information as local variables is theoretically possible but impossibly tedious. We observe that local proofs for many of these programming patterns can be achieved by exploiting *permissions* that are redistributed by atomic actions and otherwise passed around the program without duplication via input and output parameters of procedures. To track permissions, we enhance the interface of yield invariants, procedures, and atomic actions with annotations that satisfy a discipline enforced by a combination of linear typing [38] over procedure bodies and logical reasoning over the transitions of atomic actions.

The formalization in this paper is the basis of a new design and implementation of the Civl verifier. We hope that Civl will serve researchers as a viable platform for experimenting with optimizations and implementation decisions.

To summarize, this paper makes the following contributions:

- It presents a core language RefPL for expressing modular proofs of refinement over structured concurrent programs. The formulation of refinement for RefPL is general and allows the user to encode verification of an arbitrary safety property as refinement verification. Furthermore, RefPL enables the construction of layered proofs [25] of safety via iterated refinement.
- A refinement proof for RefPL is modular and decomposed along program syntax through the use of yield invariants. The interfaces to procedures, actions, and yield invariants exploit a linear typing discipline [38] that enhances local verification through the use of permissions.
- Finally, we present a robust implementation of the refinement rule and yield invariants in the Civl verifier.

### Related Work

Formal verification techniques based on stepwise refinement have long been advocated, in theory, for construction of verified programs (e.g., [5, 35, 36]). This paper takes its inspiration from TLA [28] and Event-B [3, 4] which popularized refinement as an approach for reasoning about a concurrent program modeled as a transition system. Recent efforts [10, 16, 17] have developed support for development of verified programs atop the foundation of refinement over transition systems. Our work develops a foundation and tool support for refinement over structured concurrent programs rather than flat transition systems. We are encouraged by broad interest in the use of automatic program simplification [12, 15] to reduce the complexity of reasoning about concurrent programs.

The technique of yield invariants is inspired by interference-free location invariants in the work of Owicki and Gries [34] and the rely specification in rely-guarantee reasoning [21]. Yield invariants attempt to import the reuse of rely specifications to location invariants. We introduce linear interfaces to encode permissions to address the practical concern of unwieldy ghost state. While permissions have been used before for encoding ownership in heap-manipulating programs [32], our encoding of permissions is different, applicable to any shared resource, and targeted specifically at noninterference reasoning.

There are other efforts to build practical verifiers for concurrent programs. Some verifiers focus on automation and target specific programming models and languages [7, 11, 20, 29]. Our verifier is just as automated but capable of targeting a variety of programming models because of the foundation of atomic actions in RefPL. Other verifiers share our focus on expressiveness by providing general and certified metatheory [22] but are less automated; our verifier attempts to increase expressiveness without sacrificing automation. None of these aforementioned verifiers focus on refinement and layered proofs.

Our work bears a superficial resemblance to proof methods [8, 23, 37] for linearizability [19]. Our work targets the general problem of safety verification. Linearizability is a specific safety property to which our method is applicable.

## Overview

In this section, we illustrate our contributions on a set of example programs. Section 2.1 presents yield invariants, Sect. 2.2 presents refinement, and Sect. 2.3 presents linear interfaces.

### Yield Invariants

**Fig. 1.**
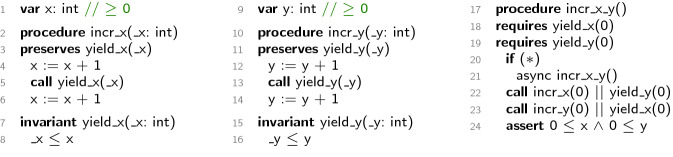
Incrementing two separate counters to illustrate yield invariants.

Figure 1 shows a simple RefPL program. The first column shows a global counter x, a procedure incr_x that increments x twice, and a yield invariant yield_x that characterizes the interference from other threads while a thread is executing incr_x. The increments of x on lines 4 and 6 are separated by a call to the yield invariant yield_x. RefPL provides a single call statement for calling any number (including zero) of procedures and yield invariants in parallel. The preserves specification on line 3 indicates that yield_x is both a precondition (usually indicated by requires) and a postcondition (usually indicated by ensures). In RefPL, each precondition of a procedure is a call to a yield invariant; all preconditions are called in parallel at procedure entry. Similarly, each postcondition is a call to a yield invariant; all postconditions are called in parallel at procedure exit.

This paper focuses on reasoning about cooperative semantics in which preemptions occur only at entry into a procedure, at a call during its execution, and at exit. The RefPL verifier proves the correctness of yield_x and incr_x modularly on these cooperative semantics. Specifically, the yield invariant yield_x is proved interference-free since the only operations in the program that modify x increment it. The procedure incr_x is proved by using the precondition of incr_x to establish the yield invariant at line 5 and then using the yield invariant to prove the postcondition at exit. This proof of incr_x depends on the observation that the input parameter _x of incr_x is passed as the argument to the three calls to yield_x: in the precondition, on line 5, and in the postcondition. The second column shows code similar to what we just discussed, except on global variable y, procedure incr_y, and yield invariant yield_y.

The third column show a procedure incr_x_y which uses recursion to create an unbounded number of concurrent threads. incr_x_y nondeterministically spawns a copy of itself on lines 20–21, calls procedures to increment x and y on lines 22–23, and asserts a safety property about x and y on line 24. Our verification goal is to prove that if a single instance of incr_x_y starts in a state that satisfies the initial constraints on x and y, indicated on lines 1 and 9 respectively, then the assertion on line 24 holds in every copy of incr_x_y.

The proof of procedure incr_x_y shows the modularity of yield invariants. First, notice that no new yield invariants are needed; the entire proof of incr_x_y is achieved by reusing yield_x and yield_y. Specifically, yield_x and yield_y are called in parallel with each other at entry, yield_y is called in parallel with incr_x at line 22, and yield_x is called in parallel with incr_y at line 23. Second, the arguments to yield_x and yield_y are specialized to match the constraints in the initial state and the assertions.

### Refining Atomic Actions

**Fig. 2.**
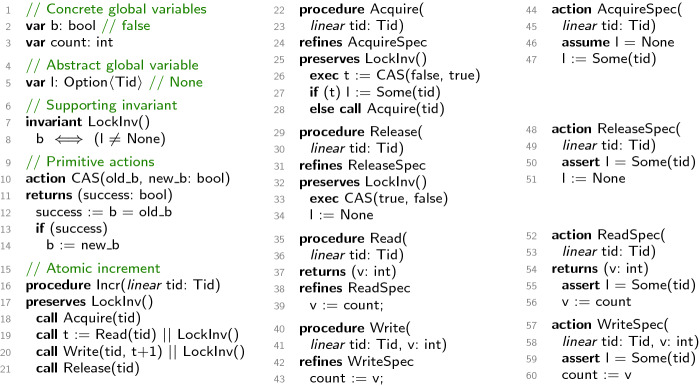
Spin lock to illustrate refinement of atomic actions.

Figure 2 shows a spin lock implementation and a client that uses the spin lock to atomically increment a shared counter. Procedure Acquire (lines 22–28) acquires the lock and procedure Release (lines 29–34) releases the lock. Both procedures use a primitive atomic action CAS (compare-and-swap) defined on lines 10–14 with two parameters—old_b and new_b. This action compares the value of a global variable b to old_b. If they are equal, b is set to new_b and true is returned, otherwise, b is not modified and false is returned. Acquire attempts to set b from false to true repeatedly via recursive call to itself (line 28) until it succeeds. Release sets b back to false from true.

Procedure Incr (lines 16–21) atomically increments the global variable count by acquiring the lock, reading count into a local variable t by calling Read (lines 35–39), writing t+1 back to count by calling Write (lines 40–43), and finally releasing the lock. We prove that Incr implements an atomic increment via a sequence of two refinement steps.

The first step abstracts the procedures Acquire, Release, Read, and Write into atomic actions AcquireSpec, ReleaseSpec, ReadSpec, and WriteSpec, respectively. These atomic actions, defined in the third column of Fig. 2, provide an explicit specification of the locking protocol for accessing the shared variable count. The specification of these actions requires the introduction of (1) a local parameter tid containing the unique id of the thread executing the code, and (2) a global variable l whose value is either None when the lock is not held or Some(tid) when the lock is held by thread tid. The second step uses these atomic actions to abstract Incr to an atomic action that increments count by 1.

There are two challenges in the first refinement proof. First, the lock implementation is defined using the concrete Boolean variable b, whereas the lock specification is defined using the logical lock variable l. Second, the implementation of Acquire is recursive, which is technically challenging for refinement reasoning. The solution to the first problem is to *introduce* l and *hide* b during the refinement proof. To introduce l into the concrete program, it is updated appropriately when Acquire (line 27) and Release (line 34) complete successfully. Furthermore, the relationship between the variables b and l is captured by the yield invariant LockInv (lines 7–8) which is used in the precondition and postcondition of Acquire and Release. The solution to the second problem is a powerful rule for refinement reasoning, described in Sect. 4, which allows the recursive call to Acquire on line 28 to be replaced by a call to the specification AcquireSpec while modularly proving that the body of Acquire refines AcquireSpec.

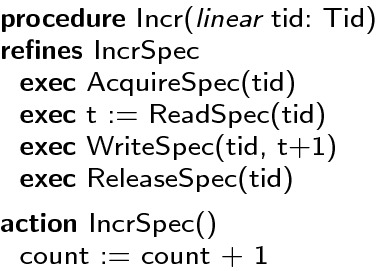



To set up the second refinement proof, the procedure calls in the body of Incr are replaced by invocations of the corresponding abstract atomic actions (as shown on the right here). The rewritten body of Incr is preemption-free; a yield may occur only at the beginning or the end. This assumption is justified by a commutativity analysis based on the observation that AcquireSpec is a right mover, ReleaseSpec is a left mover, and ReadSpec and WriteSpec are both movers [14]. Proving these mover types requires that the tid input parameters of two concurrent actions are distinct, which is specified by the *linear* annotation. In addition to encoding distinctness of values, linear variables can be used for encoding disjointness of permissions associated with values. We present an example illustrating permissions in Sect. 2.3 and a detailed technical description in Sect. 4.

For the prove that procedure Incr refines the action IncrSpec, which increments count atomically, we do not need the invariant LockInv anymore; in fact we do not need any invariant. Furthermore, the local parameter tid and the global variable l are no longer needed in the program and can be hidden. Hiding local variables is a novel feature of the refinement method described in this paper. The capability to introduce and subsequently hide global and local variables allows us to chain a sequence of refinement steps, localizing the use of variables to the parts of the proof that need them.

### Linear Interfaces

**Fig. 3.**
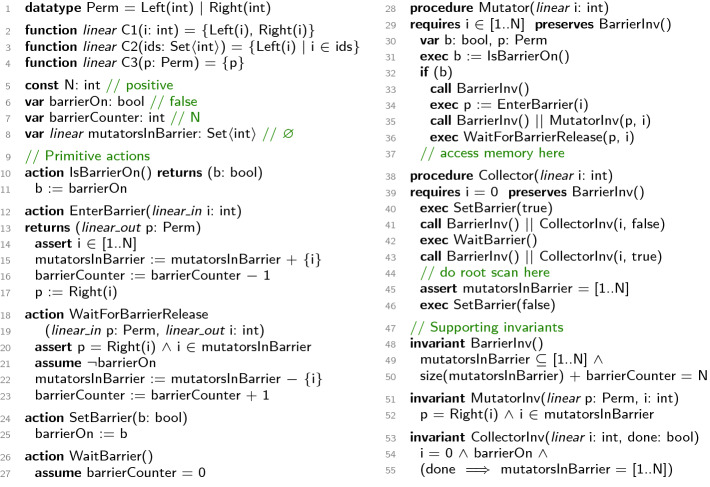
Barrier synchronization to illustrate linear interfaces.

Figure 3 shows a synchronization protocol extracted from a verified concurrent garbage collector [18]. There are N mutator threads (procedure Mutator on line 28) numbered from 1 to N, and one collector thread (procedure Collector on line 38) with ID 0. The protocol ensures that no mutator accesses memory (line 37) concurrently while the collector is doing a root scan (line 44) using barrier synchronization. Before the collector runs, it sets the Boolean variable barrierOn to true (line 40) and waits until the integer variable barrierCounter gets 0 (line 42). Before a mutator accesses memory, it reads barrierOn (line 31). If false, the mutator goes ahead. Otherwise, it signals to the collector by decrementing barrierCounter (line 34) and waits for barrierOn to be reset to false (line 36).

This example declares both global and local *linear variables* (specified by *linear*, *linear_in*, *linear_out*). Every linear variable—or more precisely, its current value—is assigned a set of *permissions* of type Perm according to the *collector functions* C1, C2, and C3. A linear integer i holds both Left(i) and Right(i), a set of integers holds the corresponding Left permissions, and a Perm value holds itself. Note that Perm is not special; any value can be a permission. For every program location we can compute the set of *available* linear variables. For example, when a mutator enters the barrier (line 34), i becomes *unavailable* because the permission Left(i) is transferred to the ghost variable mutatorsInBarrier. Then i becomes available again after exiting the barrier (line 36). Global linear variables (mutatorsInBarrier here) are always available. Parameterized by the linear collectors, our linearity framework establishes the generic invariant that all permissions across all available linear variables are disjoint. Now suppose that some mutator i is at line 37, where it holds both of its permissions and in particular Left(i), while the collector is at line 45, where mutatorsInBarrier holds all Left permissions and in particular Left(i). This situation is impossible, since the linearity feature of RefPL ensures that a duplication of permissions is impossible.

The strength of linearity, which leads to a less tedious verification task, is that its invariant connects variables from different scopes, without the need to explicitly state (and prove) this invariant. The programmer only provides a linearity specification which is checked automatically (see Sect. 4). The resulting guarantees can then be assumed “for free”. In contrast, even stating a corresponding invariant requires the introduction of auxiliary global variables and helper invariants to connect them to local variables.

## RefPL: Syntax and Semantics

In this section we present RefPL, a core programming language which is carefully designed to be (1) a minimal yet general modeling language to express concurrent programs, (2) able to express invariants over program executions, and (3) suitable for expressing (refinement-based) program transformations. RefPL focuses on interfaces for modular verification, while abstracting from detailed expression syntax and types.

**Fig. 4.**
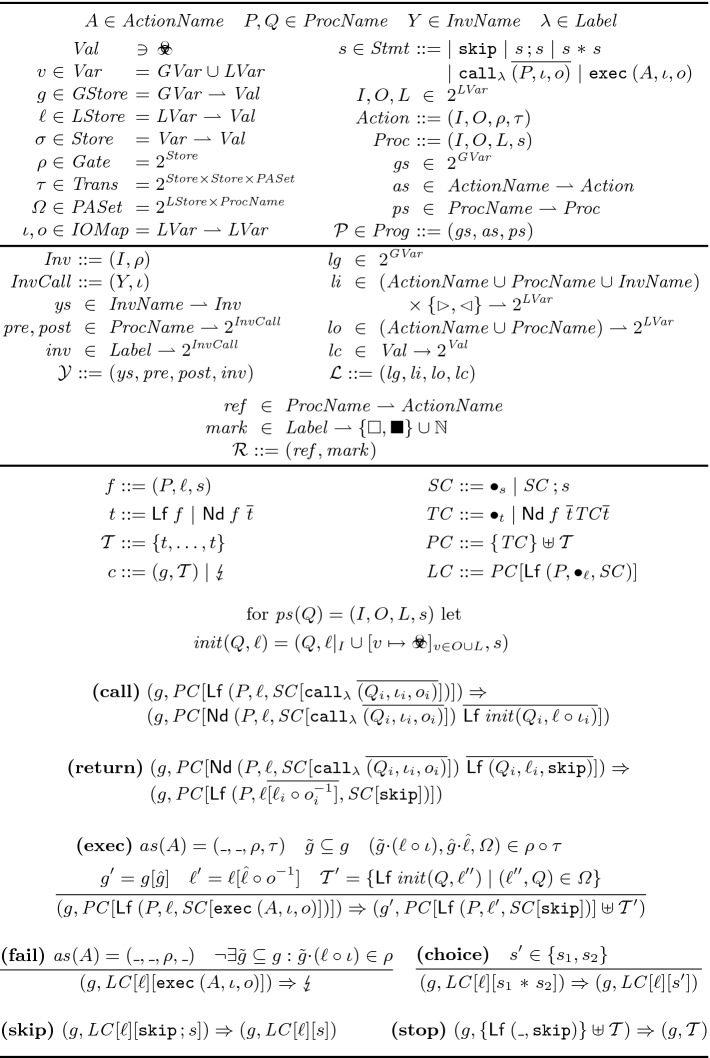
The programming language RefPL: syntax (top panel), proof annotations (middle panel), and operational semantics (bottom panel).

**Syntax.** Figure 4 (top panel) summarizes the syntax of RefPL. We assume sets of *names* which we use to name actions ($A$), procedures ($P,Q$), yield invariants ($Y$), and statement labels ($\lambda$). A set of *variables* is partitioned into *global* and *local variables*, and a *store* $\sigma$ is a partial map from variables to *values*. We write $\sigma ' \subseteq \sigma$ if $\sigma$ is an extension of $\sigma '$, $\sigma |_V$ for the restriction of $\sigma$ to *V*, $\sigma [\sigma ']$ for the store that is like $\sigma '$ on ${{\,\mathrm{dom}\,}}(\sigma ')$ and otherwise like $\sigma$, and $g\cdot \ell$ for the combination of a *global* and *local store*. A *program* consists of a finite set of global variables $gs$, a partial map $as$ from action names to actions, and a partial map $ps$ from procedure names to procedures. Both actions and procedures have an interface of *input variables* $I$ and *output variables* $O$, and procedures have additional *local variables* $L$. A *(gated atomic) action* [13, 26] consists of a *gate* $\rho$ and a *transition relation* $\tau$. The gate is a set of stores (i.e., a predicate) over $gs \cup I$. Executing the action in a state that does not satisfy the gate fails the execution. Otherwise, every transition $(\sigma ,\sigma ',\varOmega )$ in $\tau$ describes a possible atomic state transition from $\sigma$ (over $gs \cup I$) to $\sigma '$ (over $gs \cup O$), together with the creation of new asynchronous threads according to a set of *pending asyncs* $\varOmega$; every pending async $(\ell ,P) \in \varOmega$ is turned into a new thread that executes procedure $P$ with input store $\ell$. A *procedure* consists of a *statement* $s$ that is composed of standard control-flow commands and two call commands: $\mathtt {exec}$ to invoke actions and $\mathtt {call}$ for the parallel invocation of multiple procedures. Every entry in the invocation sequence of a $\mathtt {call}$ is called an *arm* of the call, and the *label* $\lambda$ is used to attach specification information to the call. Parameter passing is expressed using an *input map* $\iota$ from the callee’s formals $I$ to the caller’s actuals $I\cup O\cup L$, and an injective *output map* $o$ from the callee’s formals $O$ to the caller’s actuals $O\cup L$. Input variables are immutable, since they are not mapped to by output maps and the variables of a procedure are not modified anywhere else. Output and local variables of a procedure are initialized to the default value . In RefPL, loops are modeled using recursion, and conditional statements are modeled using nondeterministic branching ($\mathtt {*}$) and actions that assume the branching condition.

**Type Checking.** For a program we require that (1) the action name in an $\mathtt {exec}$ statement is in ${{\,\mathrm{dom}\,}}( as )$, (2) the procedure names in a $\mathtt {call}$ statement are in ${{\,\mathrm{dom}\,}}( ps )$, and the actual outputs of all arms are disjoint from each other and all actual inputs, and (3) for every pending async $(\ell ,P)$ in the transition relation of an action in ${{\,\mathrm{img}\,}}( as )$, $P\in {{\,\mathrm{dom}\,}}( ps )$ and ${{\,\mathrm{dom}\,}}(\ell )$ contains all inputs of $P$.

**Semantics.** Figure 4 (bottom panel) presents the operational semantics of RefPL, a transition relation $$\Rightarrow $$ over *configurations* that consist of a global store over $$ gs $$ and a finite multiset of threads. Each thread is a tree (which generalizes a call stack); a $$\mathtt {call}$$ statement creates new leaf nodes (Lf) and blocks the caller in an internal node (Nd) until all arms of the parallel call finish. Each tree node contains a *frame* $$(P, \ell , s)$$ that represents the current state of a procedure $$P$$ during execution: $$\ell $$ is the procedure’s current local store and $$s$$ is a statement that remains to be executed. In the definition of $$\Rightarrow $$ we use several evaluation contexts that have a unique hole $$\bullet $$; filling the hole is denoted by $$\cdot [\cdot ]$$. In particular, $$ SC [s]$$ is a statement with $$s$$ in evaluation position, and $$ PC [t]$$ is a multiset of thread trees where $$t$$ is a subtree in one of these trees. The operator $$\circ $$ means function or relation composition.

Atomic actions (invoked through the $\mathtt {exec}$ command) execute directly in the context of the caller; inline, if you will. If the current store does not satisfy the gate of an executed action, the execution stops in the *failure configuration* . It is important to appreciate the generality of atomic actions. First, they can represent atomic operations at an arbitrary level of granularity, from fine-grained low-level operations (e.g., as implemented in hardware) to coarse-grained summaries (e.g., obtained as part of a layered proof). Second, the notion of pending asyncs subsumes the need for a dedicated asynchronous call statement, and enables advanced proof techniques for asynchronous programs [24, 26]. Finally, all accesses to global variables are confined to atomic actions.

We distinguish between the *preemptive semantics* and the *cooperative semantics* of a program. The preemptive semantics $\Rightarrow$ defines the standard fine-grained behaviors of a concurrent program, where a context switch can happen at any time. A program should be proved correct under its preemptive semantics. However, for reasoning purposes we consider a cooperative semantics, where context switches only happen at procedure calls and returns. We call these locations *yields*. The justification for reducing reasoning about preemptive semantics to cooperative semantics is outside the scope of this paper (Civl uses commutativity reasoning and a reduction argument).

A leaf node $\mathsf {Lf}\, (P,\_,s)$ is *yielding*, if it denotes the *entry* or *exit* of procedure $P$, i.e., if $ps (P) = (\_,\_,\_,s)$ or $s= \mathtt {skip}$. A configuration is *yielding* if all leaves are yielding, and *cooperative* if at most one leaf is not yielding. Then the cooperative semantics is given by restricting $\Rightarrow$ to cooperative configurations. Notice that the configuration after an $\mathtt {exec}$ might be non-yielding. Thus, under cooperative semantics the pending asyncs created by $\mathtt {exec}$ can only start executing once the caller reaches the next yield. We note that arbitrary yields can be modeled with “empty” parallel calls (i.e., a $\mathtt {call}$ with no arms).

A *yield-to-yield fragment* $\{P\,|\,\kappa _1\}\, \overline{e}\, \{\kappa _2\}$ of a procedure $P$ is any sequence of $\mathtt {exec}$ statements $\overline{e}$ that forms a path in $P$ from $\kappa _1$ to $\kappa _2$, where $\kappa _1$ and $\kappa _2$ are either $\mathtt {call}$ statements, $\bot$, or $\top$ ($\kappa _1=\bot$ for procedure entries; $\kappa _2=\top$ for procedure exits). For example, procedure Acquire in Fig. 2 has three yield-to-yield fragments: (A1) entry/successful CAS/then branch/exit, (A2) entry/failed CAS/call in the else branch, and (A3) call in the else branch/exit (i.e., an “empty” fragment). Let $Gate (\overline{e})$ be the set of stores from which executing $\overline{e}$ cannot fail, and let $Trans (\overline{e})$ be the set of tuples $(\sigma , \sigma ', \varOmega )$ where executing $\overline{e}$ from store $\sigma$ can result in $\sigma '$ with all created pending asyncs collected in $\varOmega$. We define a reduced transition relation $\Rrightarrow$ over yielding configurations, such that $c\Rrightarrow c'$ if and only if there are cooperative but non-yielding configurations $(c_i)_{1\le i\le n \wedge n \ge 0}$ with $c\Rightarrow c_1 \Rightarrow \dots \Rightarrow c_n \Rightarrow c'$. Thus, every step in $\Rrightarrow$ corresponds to the execution of a yield-to-yield fragment under cooperative semantics.

## Abstracting RefPL Programs

This section presents a proof rule for transforming a concurrent program $\mathcal {P}$ into a concurrent program $\mathcal {P}'$ such that there is a simulation between the cooperative executions of $\mathcal {P}$ and $\mathcal {P}'$. The transformation comprises *variable hiding* ($\mathcal {P}'$ has fewer global and local variables than $\mathcal {P}$) and *procedure abstraction* (procedures in $\mathcal {P}$ are summarized to atomic actions in $\mathcal {P}'$). Our proof rule takes as input a *yield specification* $\mathcal {Y}$, a *linearity specification* $\mathcal {L}$, and a *refinement specification* $\mathcal {R}$ (see Fig. 4), and decomposes the refinement verification problem as follows. 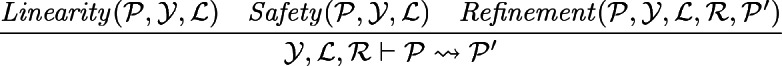


The yield specification declares yield invariants and attaches them to program locations, and the linearity specification declares linear interfaces and sets up a permission discipline (Sect. 4.1). The $Linearity$ judgment (Sect. 4.2) ensures that the linear interfaces of procedures, actions, and invariants in $\mathcal {P}$ are valid, which establishes a linear disjointness property. The $Safety$ judgment (Sect. 4.3) ensures that preconditions, postconditions, and invariants in $\mathcal {P}$ are valid and interference-free, which captures reachability information in $\mathcal {P}$. Note that $Linearity$ and $Safety$ interact, as yield invariants can have a linear interface and safety checking assumes the guarantees of linearity checking. In our proof rule, the guarantees of $Linearity$ (Lemma 1) and $Safety$ (Lemma 2) establish the context for refinement checking. However, we stress that these guarantees are useful on their own, independent of refinement. The refinement specification (Sect. 4.4) declares how $\mathcal {P}$ is converted to $\mathcal {P}'$, and the $Refinement$ judgment ensures that every execution of $\mathcal {P}$ is simulated by an execution of $\mathcal {P}'$ (Theorem 1). In Sect. 5 we show how all of our obligations are implemented in practice.

### Yield Invariants and Linear Interfaces

RefPL supports *yield invariants* of the form $$(I,\rho )$$, where $$I$$ are input variables and $$\rho $$ is a gate over $$ gs \cup I$$. In a yield specification $$\mathcal {Y}= ( ys , pre , post , inv )$$, the map $$ ys $$ assigns invariant names to yield invariants, such that invariants can be “invoked” by name—similar to actions and procedures—by supplying an input map $$\iota $$. We will write $$\varphi $$ and $$\psi $$ for sets of such *invariant calls*, and $$\sigma \models \varphi $$ to denote that store $$\sigma $$ satisfies $$\varphi $$, i.e., $$ g\cdot \ell \models \varphi \iff \forall (Y,\iota ) \in \varphi \ \exists \hat{g}\subseteq g: \hat{g}\cdot (\ell \circ \iota ) \in ys (Y).\rho $$. Then invariant calls are assigned to program locations as follows: $$ pre (P)$$ are the *preconditions* that must hold on entry to procedure $$P$$, $$ post (P)$$ are the *postconditions* that must hold on exit from procedure $$P$$, and $$ inv (\lambda )$$ are the invariants that must hold at calls labeled with $$\lambda $$. These are the yield locations in the cooperative semantics, under which we will show the invariants correct and stable under interference.

RefPL supports *linear permissions* to enhance local reasoning. The core idea of linearity is to identify a subset of *(linear) available variables* among all variables in all frames of a configuration. Every value stored in an available variable is mapped to a set of values called *permissions*, with the desired property that the values in available variables are mapped to disjoint permissions. This disjointness property can then be used as free assumption in other verification conditions.

In a linearity specification $$\mathcal {L}= ( lg , li , lo , lc )$$, the *linear global variables* $$ lg $$ are a subset of $$ gs $$, which are always available. For every action/procedure/invariant name *X*, $$ li (X,\rhd )$$ and $$ li (X,\lhd )$$ are subsets of its input variables called *linear-in* and *linear-out*, respectively. The linear-ins expect to receive from an available actual parameter, while the linear-outs ensure that their actual parameter will be available upon return. An input variable can be both linear-in and linear-out (which we assume for all invariants). For every action/procedure name *X*, its *linear outputs* $$ lo (X)$$ are a subset of its output variables, such that the receiving actual return parameters become available when *X* returns. For example, in Fig. 3 the global variable mutatorsInBarrier is linear, procedure Mutator and yield invariant CollectorInv have a linear (linear-in and linear-out) input i, action EnterBarrier has linear-in input i and linear output p, and WaitForBarrierRelease has a linear-in input p and linear-out input i. The permissions assigned to an available variable are determined by a *linear collector* function $$ lc $$, which is a flexible mechanism to encode various permission disciplines. For convenience, we lift $$ lc $$ to collect all permissions of a set of variables *V* in store $$\sigma $$, i.e., $$ \textstyle lc (\sigma , V) = \biguplus _{v\in V} lc (\sigma (v)) $$. A simple example of a collector function that expresses unique identifiers (as needed in Fig. 2) would return the singleton set $$\{\textsf {tid}\}$$ for a thread identifier variable $$\textsf {tid}$$. Figure 3 shows a more advanced usage, where the definition of $$ lc $$ is split across the functions C1, C2, and C3 (see Sect. 2.3).

### Linearity

Let us assign to every (sub)statement $$s$$ in $$\mathcal {P}$$ a *linear type* , written as , where / is the set of local variables available directly before/after executing $$s$$. Based on the linear interfaces in $$ li $$ and $$ lo $$, the most general linear types can be inferred, but for simplicity we assume all types to be given and define a type checker below. Since linear types annotate each program location with available variables, we can define the collection of linear permissions over a configuration $$c= (g, \mathcal {T})$$ as , where  ranges over all frames in all nodes of $$\mathcal {T}$$. Then the *linear disjointness property* for a configuration $$c$$ is $$ IsSet ( lc (c))$$, where $$ IsSet (\cdot )$$ states that a multiset does not contain duplicates. We call such a configuration $$\mathcal {L}$$*-valid*. The $$ Linearity (\mathcal {P},\mathcal {Y},\mathcal {L})$$ judgment comprises a semantic check on actions and a syntactic check on procedures, which ensures the preservation of the linear disjointness property as follows.

#### Lemma 1

Let $c$ be an $\mathcal {L}$-valid configuration of $\mathcal {P}$. If $c\Rightarrow c'$ then $c'$ is $\mathcal {L}$-valid.

Essentially, an execution starts with a set of permissions and redistributes these in every step. The permissions can stay the same or decrease, but never increase.

**Linear Action Checking.** All state updates (other than parameter passing) are confined to atomic actions. We need to ensure that the outgoing permissions of an action are always a subset of the incoming permissions. Thus, for every $A\in {{\,\mathrm{dom}\,}}( as )$ with $as (A) = (\_, \_, \rho , \tau )$ we check

$$\begin{aligned} (g\cdot \ell , g'\cdot \ell ', \varOmega ) \in \rho \circ \tau \wedge inPerm = \big ( lc (g, lg ) \uplus lc (\ell , li (A, \rhd ))\big ) \wedge IsSet (inPerm) \implies \\ \textstyle \Big ( lc (g', lg ) \uplus lc (\ell , li (A, \lhd )) \uplus lc (\ell ', lo (A)) \uplus \big (\biguplus _{(\ell '', P) \in \varOmega } lc (\ell '', li (P, \rhd ))\big ) \Big ) \subseteq inPerm . \end{aligned}$$

Starting with a set of permissions in the linear globals and linear-in inputs, the action can redistribute these permissions among the linear globals, its linear-out inputs and linear outputs, and the linear-ins of pending asyncs, but permissions cannot appear out of thin air. Notice that this check depends on the user-provided linear collector function $lc$. For example, consider action EnterBarrier in Fig. 3. The linear-in input i holds the permissions Left(i) and Right(i) on entry (cf. collector C1). By adding i to mutatorsInBarrier we hand over the permission Left(i) (cf. collector C2), and by the assignment to the linear output p we hand over the permission Right(i) (cf. collector C3). Thus, the set of permissions in mutatorsInBarrier and i before is the same as the permissions in mutatorsInBarrier and p after executing EnterBarrier.

**Linear Type Checking.** Now that we can trust the linear interfaces of actions, we need to ensure that the linear types in procedures “add up” w.r.t. control flow and parameter passing. For every $P\in {{\,\mathrm{dom}\,}}( ps )$ with body  we require , , and a derivation of  according to the rules in Fig. 5, where $\iota (V)$ means $\biguplus _{v\in V}\iota (v)$. For example, in procedure Mutator in Fig. 3 the linear input parameter i becomes unavailable at line 34, where it is passed as linear-in. However, this call makes the local variable p available, such that it can be passed as linear-in to the call on line 36. This call also passes i as linear-out input, which makes i available again on line 37.

**Fig. 5.**
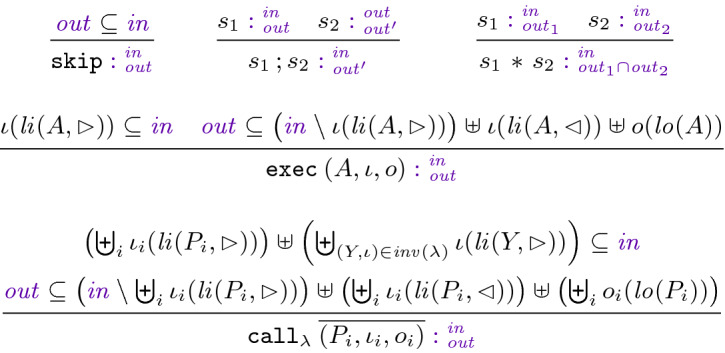
Linear type checking.

### Safety

In a yielding configuration $(g, \mathcal {T})$, every frame $(P,\ell ,s)$ in $\mathcal {T}$ is associated with a set of invariant calls $\varphi$ as follows: $\varphi = pre (P)$ if $s$ is the entry of $P$, $\varphi = post (P)$ if $s$ is $\mathtt {skip}$ (the exit of $P$), or $\varphi = inv (\lambda )$ if $s$ is blocked at a call labeled with $\lambda$. If $g\cdot \ell \models \varphi$ holds in every frame, then we call the configuration $\mathcal {Y}$*-valid*. To show that this property is preserved across the execution of a yield-to-yield fragment (i.e, a step in $\Rrightarrow$), the $Safety (\mathcal {P}, \mathcal {Y}, \mathcal {L})$ judgment is decomposed into two kinds of procedure-modular verification conditions: (1) a *sequential check* which ensures that the next $\varphi$ in the executing frame is established, and (2) a *noninterference check* which ensures that the $\varphi$’s in all other frames are preserved. Both checks weave in linearity to enhance local reasoning.

#### Lemma 2

Let $c$ be an $\mathcal {L}$-valid, $\mathcal {Y}$-valid configuration of $\mathcal {P}$. If $c\Rrightarrow c'$ then $c'$ is $\mathcal {Y}$-valid.

**Floyd Packages.** For convenience, let $pre (\kappa )$ be the set of all invariants and preconditions of a $\mathtt {call}$ statement $\kappa$ (and $post (\kappa )$ analogously):

$$\begin{aligned} pre (\mathtt {call}_{\lambda }\, \overline{(Q_i, \iota _i, o_i)})&= \textstyle inv (\lambda ) \cup \big (\bigcup _i \{(Y, \iota _i \circ \iota ) \mid (Y, \iota ) \in pre (Q_i)\} \big )\\ post (\mathtt {call}_{\lambda }\, \overline{(Q_i, \iota _i, o_i)})&= \textstyle inv (\lambda ) \cup \big (\bigcup _i \{(Y, (\iota _i\cup o_i) \circ \iota ) \mid (Y, \iota ) \in post (Q_i)\}\big ) \end{aligned}$$

For every yield-to-yield fragment $\{P\,|\,\kappa _1\}\, \overline{e}\, \{\kappa _2\}$ of $P\in {{\,\mathrm{dom}\,}}( ps )$ we define a *Floyd package* $\{P\,|\,\varphi \,|\, ll \}\, \overline{e}\, \{\psi \}$, which contains the invariants $\varphi$ and linear available variables $ll$ before, and the invariants $\psi$ after the yield-to-yield fragment:**Sequential Checking.** For every Floyd package $\{P\,|\,\varphi \,|\, ll \}\, \overline{e}\, \{\psi \}$ we checkAfter  executing $\overline{e}$ from a store with  disjoint permissions that  satisfies $\varphi$, it must be the case that  $\psi$ and  the preconditions of all created pending asyncs hold. Notice that we can assume all gates of atomic actions when executing $\overline{e}$. This is the case because yield invariants are not supposed to be strong enough to prove $\mathcal {P}$ safe. Their purpose is to establish the context for refinement checking.

**Noninterference Checking.** For every Floyd package $\{P\,|\,\varphi \,|\, ll \}\, \overline{e}\, \{\psi \}$ and every yield invariant $Y\in {{\,\mathrm{dom}\,}}( ys )$ we checkAfter  executing $\overline{e}$ from a store with  disjoint permissions that  satisfies both $\varphi$ and $Y$, it must be the case that  $Y$ still holds. A key ingredient that makes our yield invariants powerful is the possibility to pass parameters to them ($\ell '$ above, which is the same before and after executing $\overline{e}$), together with the possibility to give invariants a linear interface to include them in the disjointness assumption  . The reuse of named, parameterized invariants that are inductive on their own facilitates ergonomic and modular proofs as well as a reduction in the number of noninterference checks compared to location invariants.

The example in Fig. 3 uses three yield invariants. BarrierInv states a global property on barrierCounter and mutatorsInBarrier, MutatorInv states a property of mutators on line 35, and CollectorInv states a property of the collector at lines 41 and 43 (notice the difference in the Boolean parameter). The linear parameters to both MutatorInv and CollectorInv are essential to prove their noninterference. For example, linearity discharges all noninterference obligations of CollectorInv w.r.t. yield-to-yield fragments in procedure Collector; there cannot be two different available variables i both holding thread identifier 0. CollectorInv is also stable across the yield-to-yield fragments in procedure Mutator: by linearity, we know that EnterBarrier cannot execute if mutatorsInBarrier holds all mutator identifiers, and WaitForBarrierRelease is blocked when barrierOn is true. As an example of a sequential check, observe that the invariants at line 41 together with barrierCounter = 0 from executing WaitBarrier imply the invariants at line 43, in particular that mutatorsInBarrier holds all mutator identifiers.

### Refinement

Recall that the goal of our proof rule is to transform a program $\mathcal {P}= ( gs , as , ps )$ into a program $\mathcal {P}' = ( gs ', as ', ps ')$. So far, we showed how the two judgments $Linearity (\mathcal {P}, \mathcal {Y}, \mathcal {L})$ and $Safety (\mathcal {P}, \mathcal {Y}, \mathcal {L})$ establish properties on executions of $\mathcal {P}$, using a linearity specification $\mathcal {L}$ and yield specification $\mathcal {Y}$. In the remainder of this section we show how the $Refinement (\mathcal {P}, \mathcal {Y}, \mathcal {L}, \mathcal {R}, \mathcal {P}')$ judgment ties together $\mathcal {P}$ and $\mathcal {P}'$ using a refinement specification $\mathcal {R}$.

Consider an execution step $c\Rrightarrow c'$ of $\mathcal {P}$. We want to say that there is a representative step $\hat{c} \Rrightarrow \hat{c}'$ in $\mathcal {P}'$. Representative means that $\hat{c}$ and $\hat{c}'$ are abstract representations of $c$ and $c'$, respectively. We capture this notion in an *abstraction mapping* $\alpha$, which maps every concrete configuration of $\mathcal {P}$ to an abstract configuration of $\mathcal {P}'$. Then the meaning of the judgment $\mathcal {L},\mathcal {Y},\mathcal {R}\vdash \mathcal {P}\rightsquigarrow \mathcal {P}'$ derived by our proof rule is expressed in the following theorem.

#### Theorem 1

Let $c$ be an $\mathcal {L}$-valid, $\mathcal {Y}$-valid configuration of $\mathcal {P}$. (1) If $c\Rrightarrow \lightning$ then $\alpha (c) \Rrightarrow \lightning$. (2) If $c\Rrightarrow c'$ then either $\alpha (c) = \alpha (c')$, $\alpha (c) \Rrightarrow \alpha (c')$, or $\alpha (c) \Rrightarrow \lightning$.

The safety of $\mathcal {P}'$ should imply the safety of $\mathcal {P}$. Thus, (1) states that any failure in $\mathcal {P}$ is preserved in $\mathcal {P}'$. And (2) states that every step in $\mathcal {P}$ is matched with a (potentially stuttering) step or failure in $\mathcal {P}'$. Hence, $\mathcal {P}'$ can fail “more often” than $\mathcal {P}$, but otherwise “behaves like” $\mathcal {P}$.

**Refinement Specification.** In a refinement specification $\mathcal {R}= ( ref , mark )$, the *refinement mapping* $ref$ is a partial map from ${{\,\mathrm{dom}\,}}( ps )$ to ${{\,\mathrm{dom}\,}}( as ')$. For every procedure $P\in {{\,\mathrm{dom}\,}}( ref )$, we check that $P$ is abstracted by action $A= ref (P)$. Since our refinement checks are procedure-modular, we require ${{\,\mathrm{dom}\,}}( ref )$ to be closed under calls in $ps$ (not including pending asyncs). In general, $P$ executes multiple yield-to-yield fragments and possibly calls other procedures, while $A$ executes in a single atomic step. Thus we need to ensure that exactly one yield-to-yield fragment in $P$ behaves like $A$, while all other fragments have no visible side effect. We use a *marking function* $mark$ to identify where $A$ should happen in $P$. For every call statement with label $\lambda$, $mark (\lambda )$ is either $\square$ (“before”), $\blacksquare$ (“after”), or the index $i\in \mathbb {N}$ of some arm of the call. This means that we are still before $A$ when the call returns, that we are already after $A$ when reaching the call, or that arm *i* establishes $A$, respectively. Naturally, procedure entry and exit are marked with $\square$ and $\blacksquare$, respectively. Then the marks along every path of $P$ must match the regular expression $\square ^+\mathbb {N}^?\blacksquare ^+$, which distinguishes two cases. (M1) No call is marked with an index $i\in \mathbb {N}$. Then some yield-to-yield fragment switches from $\square$ to $\blacksquare$, which we will check to behave like $A$. All other yield-to-yield fragments and calls on the path must have no side effect. (M2) Some call is marked with index $i\in \mathbb {N}$. We will check that arm *i* of this call behaves like $A$, while all other calls and yield-to-yield fragments on the path must have no side effect. Since we check $mark$ per path, there are in general multiple occurrences of (M1) and (M2).

In Fig. 2, the $ref$ mapping is specified using the refines keyword. For example, procedure Acquire refines the atomic action AcquireSpec. The $mark$ mapping is not explicitly specified, but we consider the call on line 28 to be marked with 1 (the index of its only arm). Then one path through Acquire is marked with $\square \blacksquare$ and the other one with $\square \,1\,\blacksquare$, both matching the regular expression above.

**Program Rewriting.** The program $$\mathcal {P}= ( gs , as , ps )$$ is rewritten into $$\mathcal {P}' = ( gs ', as ', ps ')$$ as follows. First, global variables can be hidden, such that $$ gs ' \subseteq gs $$. Second, new atomic actions can be added (for new abstractions of procedures) and unreferenced ones removed, but for $$A\in {{\,\mathrm{dom}\,}}( as )\cap {{\,\mathrm{dom}\,}}( as ')$$ we require $$ as '(A) = as (A)$$. Recall that an action can execute in any program that contains the referenced global variables and procedures. Third, $${{\,\mathrm{dom}\,}}( ps ') = {{\,\mathrm{dom}\,}}( ps )$$ and we rewrite every $$ ps (P) = (I,O,L,s)$$ into $$ ps '(P) = (I',O',L',s')$$ as follows. Local variables can be hidden, such that $$I' \subseteq I\wedge O' \subseteq O' \wedge L' \subseteq L$$. If $$P\not \in {{\,\mathrm{dom}\,}}( ref )$$, then $$s'$$ is like $$s$$, except that call arms $$(Q, \iota , o)$$ with $$ ps '(Q) = (I_Q, O_Q, \_, \_)$$ turn into $$(Q, \iota |_{I_Q}, o|_{O_Q})$$, with the requirement $${{\,\mathrm{img}\,}}(o) \cap (O' \cup L') = {{\,\mathrm{img}\,}}(o|_{O_Q})$$ that formal and actual outputs can only be hidden together. We denote this rewriting of a statement by $$\alpha (s)$$. If $$P\in {{\,\mathrm{dom}\,}}( ref )$$, then $$s' = \mathtt {exec}\, ( ref (P), id (I'), id (O'))$$, where $$ id (\cdot )$$ is the identity mapping on a given set of variables. We denote this $$\mathtt {exec}$$ statement by $$\alpha (P)$$. Thus, procedures in $${{\,\mathrm{dom}\,}}( ref )$$ remain in $$\mathcal {P}'$$, but with their bodies rewritten to a single $$\mathtt {exec}$$ to their abstraction. Clearly, the action interface $$ as '\circ ref (P) = (I',O',\_,\_)$$ must match the procedure, and $$L' = \varnothing $$. Overall, $$\mathcal {P}'$$ must still typecheck, which ensures, e.g., that the remaining actuals in input/output maps were not hidden.

In the first refinement step of Sect. 2.2, where the procedures in the second column of Fig. 2 are abstracted to the atomic actions in the third column, the global variable b is hidden. In the second refinement step, where procedure Incr is abstracted to action IncrSpec, the input parameter tid and the global variable l are hidden. Notice that, in order to chain together these two refinement steps, we performed an auxiliary rewriting step in procedure Incr that converted $\mathtt {call}$ statements to $\mathtt {exec}$ statements. Civl automatically performs this transformation as part of a refinement step, justified by a commutativity argument we explained in Sect. 2.2. However, this rewriting is not formalized as part of our refinement rule in this paper.

**Skip Action.** In the following we assume a special action $Skip$ that has no inputs and outputs, does not modify global variables, and creates no pending asyncs. Formally, $as ( Skip ) = (\varnothing , \varnothing , \{\varepsilon \}, \{(\varepsilon ,\varepsilon ,\varnothing )\})$, where $\varepsilon$ is the empty store. Observe that safety verification (i.e., showing that the failure configuration $\lightning$ is unreachable) is a special case of refinement, where all global and local variables are hidden, and all procedures are abstracted to $Skip$.

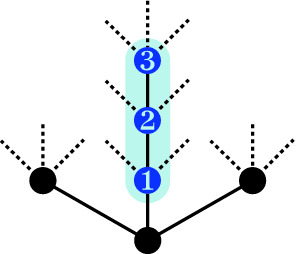



**Abstraction Mapping.** Figure 6 defines the abstraction mapping $$\alpha $$. In a given yielding configuration, we restrict the global store to $$ gs '$$ and drop all trees rooted in a node that refines $$ Skip $$. The remaining nodes are traversed recursively, where frames with $$P\not \in {{\,\mathrm{dom}\,}}( ref )$$ (nodes $$\bullet $$ on the right) are rewritten as expected. The interesting case is for nodes with $$P\in {{\,\mathrm{dom}\,}}( ref )$$, like node  on the right. In this case,  is turned into a leave (cutting off the remaining subtree) whose statement is either $$\alpha (P)$$ (the single $$\mathtt {exec}$$ of $$ ref (P)$$) or $$\mathtt {skip}$$. Intuitively, to match the concrete steps of $$P$$ (in  and its subnodes), the abstract configuration first stutters at $$\alpha (P)$$, then transitions to $$\mathtt {skip}$$ when the effect of $$ ref (P)$$ happens, and then stutters at $$\mathtt {skip}$$ until the return from  . The delicate part is to determine if $$ ref (P)$$ happened and to compute the local store for the abstract configuration. This is done by the *early-return function* $$r$$. The function recurses on the unique path of marked arms in calls,  in our example, and either returns $$\square $$ (when “before $$ ref (P)$$”) or a local store $$\ell $$ (when “after $$ ref (P)$$”). Suppose that  ,  ,  have local stores $$\ell _1,\ell _2,\ell _3$$, and that . Then  equals $$\ell _2$$ updated with the return parameters from $$\ell _3$$, say $$\ell _2'$$, and similarly  equals $$\ell _1$$ updated with the return parameters from $$\ell _2'$$, say $$\ell _1'$$, which is the local store for the abstract configuration. Thus, $$r$$ performs “early” return parameter passing, even though we are still in the middle of executing procedures. To prove Theorem 1, our verification conditions below have to ensure that throughout subsequent concrete execution steps,  remains $$\ell _1'$$.

**Fig. 6.**
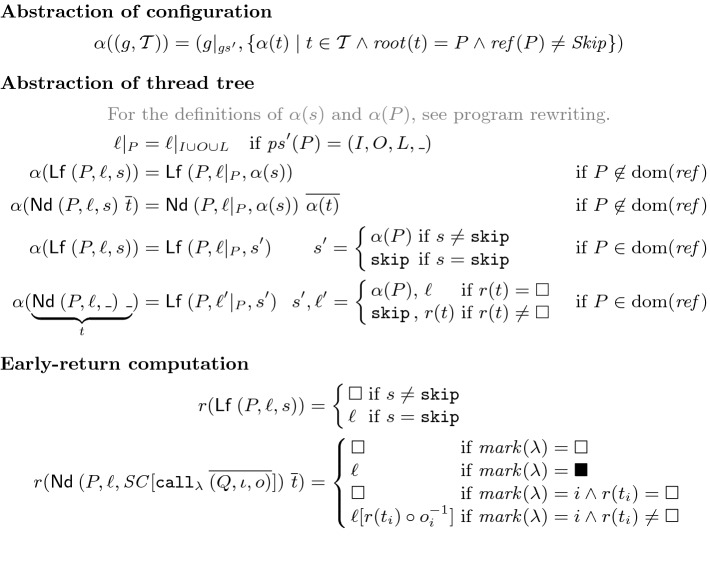
Abstraction mapping from configurations of $\mathcal {P}$ to configurations of $\mathcal {P}'$.

**Refinement Packages.** In a procedure $$P\in {{\,\mathrm{dom}\,}}( ref )$$, the effect of the abstract action $$ ref (P)$$ can happen either in a yield-to-yield fragment directly in $$P$$, or nested inside another called procedure. To handle (potentially recursive) procedure calls during refinement, we decompose the problem into procedure-modular checks. Recall that the marking function $$ mark $$ identifies yield-to-yield fragments and call arms in $$P$$ that should behave like the abstract action $$ ref (P)$$. Conversely, all other yield-to-yield fragments and call arms should have no side effect, which is to say that they should behave like $$ Skip $$. Hence we have a refinement obligation for *every* yield-to-yield fragment and *every* call arm in $$P$$, where refinement is either checked against $$ ref (P)$$ or $$ Skip $$. We capture all these refinement obligations uniformly in *refinement packages* of the form $$\{P\,|\,\varphi \,|\, ll \}\, \overline{e}\, \{A\}$$, where $$P$$ is the procedure we check refinement for, $$\varphi $$ is a set of invariant calls and $$ ll $$ a set of available variables we can assume, $$\overline{e}$$ is an $$\mathtt {exec}$$ sequence denoting the effect we check refinement for, and $$A$$ is the action we check refinement against.

*(R1) Refinement Packages for Yield-to-Yield Fragments.* For every procedure $$P\in {{\,\mathrm{dom}\,}}( ref )$$ and yield-to-yield fragment $$\{P\,|\,\kappa _1\}\, \overline{e}\, \{\kappa _2\}$$ of $$P$$ we define the refinement package $$\{P\,|\,\varphi \,|\, ll \}\, \overline{e}\, \{A\}$$ where $$\varphi $$ and $$ ll $$ are defined the same as for Floyd packages, and $$A= ref (P)$$ if $$ mark (\kappa _1)=\square $$ and $$ mark (\kappa _2)=\blacksquare $$, or $$A= Skip $$ otherwise. This case is rather straightforward. We proved the validity of $$\varphi $$ and $$ ll $$ before the fragment, and need to check that the code $$\overline{e}$$ in the fragment behaves either like $$ ref (P)$$ or $$\mathtt {skip}$$.

*(R2) Refinement Packages for Call Arms.* For every procedure $$P\in {{\,\mathrm{dom}\,}}( ref )$$ and  in $$P$$, let $$\varphi = inv (\lambda )$$ and . At a call we know the validity of the invariants attached to the call and the availability of  minus the linear variables passed into the callees. Then for every arm $$(Q_i, \iota _i, o_i)$$, let $$A_i = ref (P)$$ if $$ mark (\lambda )=i$$ or $$A_i = Skip $$ otherwise. Now the final missing ingredient for a refinement package $$\{P\,|\,\varphi \,|\, ll \}\, \overline{e}\, \{A_i\}$$ for every arm *i* is the effect $$\overline{e}$$ for which we check refinement against $$A_i$$. To obtain a modular check, our solution is to use the abstract action specification of the callee $$Q_i$$. Formally, $$\overline{e}= \mathtt {exec}\, (B_i,\iota _i|_I,o_i|_O)$$ for $$B_i = ref (Q_i)$$ with $$ as '(B_i) = (I,O,\_,\_)$$. Recall that this is well-defined, since $${{\,\mathrm{dom}\,}}( ref )$$ is closed under calls. Notice that using the specification of a callee while checking the specification of a caller is akin to reasoning with procedure pre- and postconditions, where circular dependencies are resolved via induction on the nesting depth.

Recall (from the end of Sect. 3) that procedure Acquire in Fig. 2 has three yield-to-yield fragments: (A1), (A2), (A3). Each fragment induces an (R1)-type refinement package, where (A1) is checked against AcquireSpec, while both (A2) and (A3) are checked against $Skip$. Furthermore, the call on line 28 induces an (R2)-type refinement package against AcquireSpec.

**Refinement Checking.** The $Refinement (\mathcal {P}, \mathcal {Y}, \mathcal {L}, \mathcal {R}, \mathcal {P}')$ judgment requires every refinement package $\{P\,|\,\varphi \,|\, ll \}\, \overline{e}\, \{A\}$ to be discharged as follows. Let $e= \mathtt {exec}\, (A, id (I), id (O))$ for $as '(A)=(I,O,\_,\_)$ be the abstract effect we check refinement against, let $V = gs '\cup I'\cup O'$ for $as '\circ ref (P) = (I',O',\_,\_)$ be the non-hidden variables in the scope of the refinement package, and check

$$\begin{aligned} \text {where } \varOmega |_ ref = \{(\ell ,Q) \in \varOmega \mid ref (Q)\ne Skip \}. \end{aligned}$$

We assume a store $g\cdot \ell$ that satisfies  invariants and  linear disjointness according to the refinement package. Then refinement consists of two parts, failure preservation and behavior preservation. First,  if $\overline{e}$ can fail in the concrete then $e$ must also fail in the abstract. Second,  if $e$ cannot fail in the abstract and $\overline{e}$ can transition to store $g'\cdot \ell '$ while creating pending asyncs $\varOmega$ in the concrete, then there must be a matching transition of $e$ in the abstract. Here matching means that $e$ starts in a store $\hat{g}\cdot \hat{\ell }$ that agrees with $g\cdot \ell$ on the non-hidden variables *V*, ends in a store $\hat{g}'\cdot \hat{\ell }'$ that agrees with $g'\cdot \ell '$ on *V*, and creates the same pending asyncs except the ones to procedures abstracted to $Skip$.

## Implementation

Civl is a refinement-based verifier for concurrent programs built on top of the widely-used Boogie intermediate verification language. The Boogie [6] verifier provides infrastructure for compiling annotated sequential procedures into logical verification conditions whose validity is checked by a satisfiability-modulo-theories solver. Civl is implemented as an extension of Boogie, which takes as input an annotated layered concurrent program [25] (in a language whose core is RefPL), performs concurrency-specific type checking and static analyses, and then encodes all the verification conditions of its proof rule into a standard sequential Boogie program. Thus, Civl can be understood as a compiler that eliminates concurrency in a RefPL program by translating it down to a collection of sequential procedures, thus reusing the rest of the Boogie pipeline unchanged.

The open-source Civl verifier is a stable tool which is part of the master branch [2] and public release [1] of Boogie. Civl has over 100 regression tests comprising both realistic programs and microbenchmarks. There are many published papers [9, 26, 27, 33, 39] that describe nontrivial examples verified using Civl, most written by researchers other than the developers of Civl. The code in Civl is extensible; entirely new tactics for rewriting concurrent programs have been added to it [24, 26]. Finally, Civl is designed for interactive program development. It is fast and provides several command-line flags to focus verification on parts of the program. Civl has fine-grained error reporting including error traces, which attributes a verification failure to a particular check, local to a small part of the program. This helps the programmer to debug and iteratively improve both implementation and specification.

An early version of the Civl verifier was reported by Hawblitzel et al. [18]. The implementation of the techniques described in this paper has been done as part of the new design and implementation of Civl based on the framework of layered concurrent programs [25]. In the rest of this section, we will continue to use Civl to refer to our new implementation. We now present an overview of the different parts of the verifier.

**Type Checking.** In addition to the standard type checking of a Boogie program, the Civl type checker performs several extra checks. First, it checks that the layer specifications [25] on program elements such as global and local variables, atomic actions, and procedures are correct. Second, it checks using a dataflow analysis that it is sufficient to reason about the safety of cooperative semantics. This analysis exploits mover type [14] annotations on atomic actions to reason that yield-to-yield code fragments satisfy the requirements of Lipton reduction [30]. It also generates logical verification conditions whose validity guarantee the correctness of the mover annotations on atomic actions.

**Linearity Checking.** The Civl linearity checker implements the method described in Sect. 4.2 in two parts. First, it creates for each atomic action a sequential procedure which verifies that the multiset of outgoing permissions is a subset of the multiset of incoming permissions. We use the generalized array theory [31] to encode multisets, and the $IsSet$ constraint in particular. Second, it type checks each procedure to compute the set of available variables at each control location and to verify that linear interfaces of called procedures and atomic actions are used appropriately.

**Safety Checking.** The Civl safety checker implements the method described in Sect. 4.3. Unlike the formal description which enumerates yield-to-yield code fragments, the implementation is efficient, encodes all code fragments in a RefPL procedure into a single sequential procedure with maximal sharing, and adds the safety checks by injecting instrumentation code and assertions into a cloned copy of the original procedure. To express the noninterference check, we add instrumentation variables that take snapshots of global and output variables at every yield. Furthermore, the generalized array theory is used here as well to record the pending asyncs created in a yield-to-yield code fragment, such that their preconditions can be checked.

**Refinement Checking.** The Civl refinement checker implements the method described in Sect. 4.4. Similar to safety checking, the refinement checks are added as instrumentation to procedure copies. At every yield, snapshot variables (similar as for noninterference) are used to refer to the state at the previous yield when asserting the appropriate transition relation. Civl computes a representation of the transition relation of an atomic actions as a logical formula from the user-provided representation as imperative code.

## Conclusions

In this paper, we provide a foundation for refining structured concurrent programs and an implementation in the Civl verifier. The contribution of this paper, and that of Civl in general, is the capability to express *new proofs* with significant advantages for the programmer in terms of proof structuring, annotation effort, and tool performance.

## References

[1] Boogie (release). https://www.nuget.org/packages/Boogie

[2] Boogie (source code). https://github.com/boogie-org/boogie

[3] Abrial J (1996). The B-Book: Assigning Programs to Meanings.

[4] Abrial, J., Butler, M.J., Hallerstede, S., Hoang, T.S., Mehta, F., Voisin, L.: Rodin: an open toolset for modelling and reasoning in Event-B. Int. J. Softw. Tools Technol. Transf. **12**(6) (2010). 10.1007/s10009-010-0145-y

[5] Back R, von Wright J (1998). Refinement calculus: a systematic introduction. Graduate Texts Comput. Sci..

[6] Barnett, M., Chang, B.E., DeLine, R., Jacobs, B., Leino, K.R.M.: Boogie: a modular reusable verifier for object-oriented programs. In: FMCO (2005). 10.1007/11804192_17

[7] Blom, S., Darabi, S., Huisman, M., Oortwijn, W.: The VerCors tool set: verification of parallel and concurrent software. In: IFM (2017). 10.1007/978-3-319-66845-1_7

[8] Bouajjani, A., Emmi, M., Enea, C., Mutluergil, S.O.: Proving linearizability using forward simulations. In: CAV (2017). 10.1007/978-3-319-63390-9_28

[9] Bouajjani, A., Enea, C., Mutluergil, S.O., Tasiran, S.: Reasoning about TSO programs using reduction and abstraction. In: CAV (2018). 10.1007/978-3-319-96142-2_21

[10] Chajed, T., Kaashoek, M.F., Lampson, B.W., Zeldovich, N.: Verifying concurrent software using movers in CSPEC. In: OSDI (2018). https://www.usenix.org/conference/osdi18/presentation/chajed

[11] Cohen, E., et al.: VCC: a practical system for verifying concurrent C. In: TPHOLs (2009). 10.1007/978-3-642-03359-9_2

[12] Damian, A., Dragoi, C., Militaru, A., Widder, J.: Communication-closed asynchronous protocols. In: CAV (2019). 10.1007/978-3-030-25543-5_20

[13] Elmas, T., Qadeer, S., Tasiran, S.: A calculus of atomic actions. In: POPL (2009). 10.1145/1480881.1480885

[14] Flanagan, C., Qadeer, S.: A type and effect system for atomicity. In: PLDI (2003). 10.1145/781131.781169

[15] von Gleissenthall, K., Kici, R.G., Bakst, A., Stefan, D., Jhala, R.: Pretend synchrony: synchronous verification of asynchronous distributed programs. In: POPL (2019). 10.1145/3290372

[16] Gu, R., et al.: Certified concurrent abstraction layers. In: PLDI (2018). 10.1145/3192366.3192381

[17] Hawblitzel, C., et al.: IronFleet: proving practical distributed systems correct. In: SOSP (2015). 10.1145/2815400.2815428

[18] Hawblitzel, C., Petrank, E., Qadeer, S., Tasiran, S.: Automated and modular refinement reasoning for concurrent programs. In: CAV (2015). 10.1007/978-3-319-21668-3_26

[19] Herlihy, M., Wing, J.M.: Linearizability: a correctness condition for concurrent objects. ACM Trans. Program. Lang. Syst. **12**(3) (1990). 10.1145/78969.78972

[20] Jacobs, B., Smans, J., Philippaerts, P., Vogels, F., Penninckx, W., Piessens, F.: VeriFast: a powerful, sound, predictable, fast verifier for C and Java. In: NFM (2011). 10.1007/978-3-642-20398-5_4

[21] Jones, C.B.: Specification and design of (parallel) programs. In: IFIP Congress (1983)

[22] Jung, R., Krebbers, R., Jourdan, J., Bizjak, A., Birkedal, L., Dreyer, D.: Irisfrom the ground up: a modular foundation for higher-order concurrentseparation logic. J. Funct. Program. **28** (2018).10.1017/S0956796818000151

[23] Khyzha, A., Dodds, M., Gotsman, A., Parkinson, M.J.: Proving linearizability using partial orders. In: ESOP (2017). 10.1007/978-3-662-54434-1_24

[24] Kragl, B., Enea, C., Henzinger, T.A., Mutluergil, S.O., Qadeer, S.: Inductive sequentialization of asynchronous programs. In: PLDI (2020). 10.1145/3385412.3385980

[25] Kragl, B., Qadeer, S.: Layered concurrent programs. In: CAV (2018). 10.1007/978-3-319-96145-3_5

[26] Kragl, B., Qadeer, S., Henzinger, T.A.: Synchronizing the asynchronous. In: CONCUR (2018). 10.4230/LIPIcs.CONCUR.2018.21

[27] Krishna, S., Emmi, M., Enea, C., Jovanovic, D.: Verifying visibility-based weak consistency. In: ESOP (2020). 10.1007/978-3-030-44914-8_11

[28] Lamport, L.: Specifying Systems, The TLA+ Language and Tools for Hardware and Software Engineers (2002)

[29] Leino, K.R.M., Müller, P., Smans, J.: Verification of concurrent programs with Chalice. In: FOSAD (2009). 10.1007/978-3-642-03829-7_7

[30] Lipton, R.J.: Reduction: a method of proving properties of parallel programs. Commun. ACM **18**(12) (1975). 10.1145/361227.361234

[31] de Moura, L.M., Bjørner, N.: Generalized, efficient array decision procedures. In: FMCAD (2009). 10.1109/FMCAD.2009.5351142

[32] Müller, P., Schwerhoff, M., Summers, A.J.: Viper: a verification infrastructure for permission-based reasoning. In: VMCAI (2016). 10.1007/978-3-662-49122-5_2

[33] Mutluergil SO, Tasiran S (2018). A mechanized refinement proof of the Chase–Lev deque using a proof system. Computing.

[34] Owicki, S.S., Gries, D.: Verifying properties of parallel programs: an axiomatic approach. Commun. ACM **19**(5) (1976). 10.1145/360051.360224

[35] de Roever, W.P., de Boer, F.S., Hannemann, U., Hooman, J., Lakhnech, Y., Poel,M., Zwiers, J.: Concurrency Verification: Introduction to Compositional and Noncompositional Methods. Cambridge Tracts in Theoretical Computer Science, vol. 54 (2001)

[36] Schneider FB (1997). On concurrent programming. Graduate Texts Comput. Sci..

[37] Vafeiadis, V.: Automatically proving linearizability. In: CAV (2010). 10.1007/978-3-642-14295-6_40

[38] Walker, D.: Substructural type systems. In: Pierce, B.C. (ed.) Advanced Topics in Types and Programming Languages, pp. 3–44. The MIT Press (2004). 10.7551/mitpress/1104.003.0003

[39] Wilcox, J.R., Flanagan, C., Freund, S.N.: VerifiedFT: a verified, high-performance precise dynamic race detector. In: PPoPP (2018). 10.1145/3178487.3178514

